# A Pilot Single Cell Analysis of the Zebrafish Embryo Cellular Responses to Uropathogenic *Escherichia coli* Infection

**DOI:** 10.20411/pai.v7i1.479

**Published:** 2022-02-04

**Authors:** Ashley Rawson, Vijay Saxena, Hongyu Gao, Jenaya Hooks, Xiaoling Xuei, Patrick McGuire, Takashi Hato, David S. Hains, Ryan M. Anderson, Andrew L. Schwaderer

**Affiliations:** 1 Indiana University School of Medicine, Department of Pediatrics, Division of Nephrology; 2 Indiana University School of Medicine, Department of Medical & Molecular Genetics; 3 Indiana University School of Medicine, Department of Medicine, Division of Nephrology; 4 University of Chicago, Section of Endocrinology, Diabetes and Metabolism

**Keywords:** Urinary tract infection, Urosepsis, Innate immunity, Zebrafish, Single cell RNAseq

## Abstract

**Background:**

Uropathogenic *Escherichia coli* (UPEC) infections are common and when they disseminate can be of high morbidity.

**Methods:**

We studied the effects of UPEC infection using single cell RNA sequencing (scRNAseq) in zebrafish. Bulk RNA sequencing has historically been used to evaluate gene expression patterns, but scRNAseq allows gene expression to be evaluated at the single cell level and is optimal for evaluating heterogeneity within cell types and rare cell types. Zebrafish cohorts were injected with either saline or UPEC, and scRNAseq and canonical pathway analyses were performed.

**Results:**

Canonical pathway analysis of scRNAseq data provided key information regarding innate immune pathways in the cells determined to be thymus cells, ionocytes, macrophages/monocytes, and pronephros cells. Pathways activated in thymus cells included interleukin 6 (IL-6) signaling and production of reactive oxygen species. Fc receptor-mediated phagocytosis was a leading canonical pathway in the pronephros and macrophages. Genes that were downregulated in UPEC vs saline exposed embryos involved the cellular response to the Gram-negative endotoxin lipopolysaccharide (LPS) and included Forkhead Box O1a *(Foxo1a)*, Tribbles Pseudokinase 3 (*Trib3)*, Arginase 2 (*Arg2)* and Polo Like Kinase 3 (*Plk3).*

**Conclusions:**

Because 4-day post fertilization zebrafish embryos only have innate immune systems, the scRNAseq provides insights into pathways and genes that cell types utilize in the bacterial response. Based on our analysis, we have identified genes and pathways that might serve as genetic targets for treatment and further investigation in UPEC infections at the single cell level.

## VISUAL ABSTRACT

**Figure:**
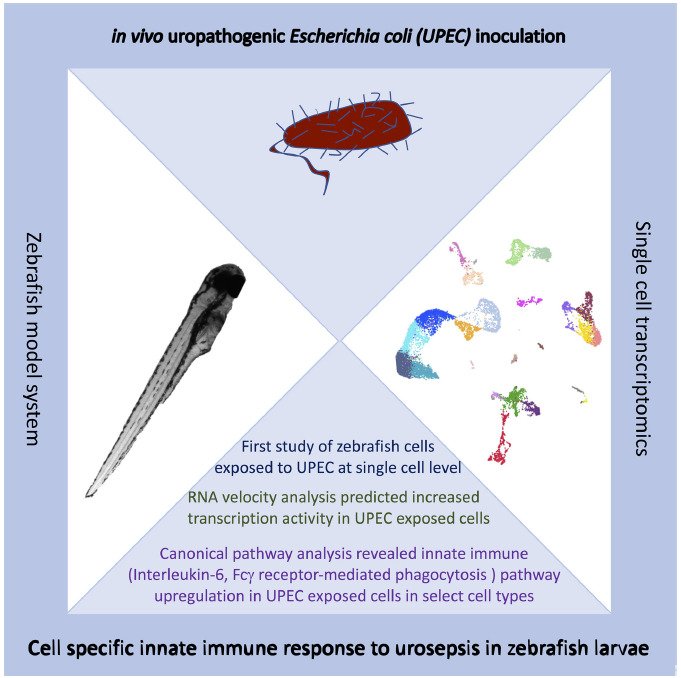

## INTRODUCTION

Urinary tract infections (UTIs) are common infections encountered in the pediatric and adult populations and are a major cause of morbidity and mortality [1, 2]. UTIs can ascend causing infection in the kidney or even progress to bacteremia or urosepsis. Urosepsis accounts for 10%-30% of septic shock episodes and uropathogenic *E.coli* (UPEC) is responsible for almost 75% of cases [3, 4]. Therefore, increased understanding of the effects of UPEC infections at the cellular level will provide the foundation for clinical care improvements.

Animal models of UTI, pyelonephritis, and urosepsis include a direct intra-renal injection of bacteria or an ascending UTI via transurethral inoculation of bacteria into rats, mice, or pigs [5–8]. These models are limited by being labor intensive and resulting in heterogenous infection burdens. Additionally use of higher vertebrates should be avoided for some research questions that might be answered by using lower vertebrate models. The zebrafish is useful for an infection model because the innate immune system is the only defense available to the zebrafish until about 4-6 weeks post fertilization [9]. Also, zebrafish may be studied at high throughput, once model systems are established. Single cell RNA sequencing (scRNAseq) represents a rapidly advancing methodology to identify and profile distinct cell types [10]. Specifically, scRNAseq can be used to identify cell types and the physiologic state of individual cells in a relatively unbiased manner [11]. Therefore, the goal of this study was to develop a model to study the effects of UPEC infection in single zebrafish cells.

## METHODS

### Ethics approval

Animal research was carried out in accordance with the National Institutes of Health guide for the care and use of Laboratory animals (NIH Publications No. 8023, revised 1978). The study was approved by the Indiana University Institutional Animal Care and Use Committee (IACUC) protocol # 11437.

## ZEBRAFISH MAINTENANCE AND EMBRYO COLLECTION

Wildtype zebrafish were maintained at 28.5°C in a recirculating aquaculture system [12]. Wild-type embryos were collected at spawning and maintained in a 28.5°C incubator in egg water-filled petri dishes [12, 13].

### Growing Red fluorescent protein-labeled UTI-89

RFP-labeled UPEC (UTI89), a cystitis strain, kindly gified to us by Dr. Molly Ingersoll (Institut Pasteur, France) was added to about 4 mL of sterile LB in an Erlenmeyer flask [14]. The flask was placed in a 37°C static incubator overnight. Then 1 mL of bacterial mixture was centrifuged to form a pellet, resuspended in sterile PBS, and diluted to an optical density (OD) of 0.073 which corresponds with ~2x10^8^ colony forming units (cfu)/mL.

### Zebrafish infection

Embryos matured for ~4 days post fertilization in a 28.5°C incubator in egg water-filled petri dishes. Embryos were separated into control and experimental groups. Then ~60 embryos were injected with UPEC and ~30 were injected with saline control. The embryos were anesthetized with tricaine and immobilized in methylcellulose. A mixture of the bacterial solution described above, phenol red, and PBS was made and loaded into a microneedle with ~1 nL of the mixture injected into the space between the epidermis and heart tube of each embryo. The control group was injected with 1 nL of phenol red and PBS. The embryos were then incubated for 24 hours at 28.5°C.

### Confirmation of infection

The embryos were then euthanized and homogenized. The homogenate of each group was then plated on an LB broth petri dish. The plate was incubated overnight at 37°C. To determine if the embryos were infact infected, we visualized the homogenate plate from each group under the Keyence All-in-One Fluorescence Microscope using BZ II Analyzer software. To identify *Escherichia coli* (*E. coli)* infection in cells, *E. coli* UTI89 genome (NC_007946.1) was added to the Zebrafish reference genome and scRNAseq was analyzed as described above.

### Embryo collection and dissociation

The control and experimental groups of ~4-day post fertilization wildtype zebrafish embryos were again injected as described above. After they were incubated for 24 hours at 28.5°C, the embryos were euthanized with tricaine and ice was added to the egg water. The embryos were added to methylcellulose and the heads of the embryos were removed using a straight blade sterile scalpel to allow increased focus on the visceral organ cells. The remaining portion of the embryos were collected and washed 2x with cold PBS at 700*g* for 5 minutes then dissociated with Liberase TL (Sigma) containing DNAse I (Sigma) in DMEM using gentleMACS dissociator (Miltenyi Biotec) in C tubes with the gentleMACS program Spleen_03. Then cells were incubated at 37°C for 10 minutes with continuous monitoring. When cell homogenate started to disappear, DMEM containing 10% FBS was added to stop the dissociation reaction. Cells were then filtered through a 70 μm filter, and centrifuged at 300g rpm for 10 minutes, counted on a hemocytometer with dead cells removed using dead cell removal microbeads and an MS column (Miltenyi Biotec). Cells were centrifuged and resuspended in DPBS (without Ca^2+^ or Mg^2+^). Final viable cells were ~4000 in saline and ~200,000 in the UPEC sample.

### Library preparation

Library preparation was performed using Chromium™ Next GEM Single Cell 3' GEM, Library & Gel Bead Kit v3.1. A single cell 3' RNAseq experiment was conducted using the Chromium single cell system (10x Genomics, Inc) and the NovaSeq 6000 sequencer (Illumina, Inc). Following cell capture and cell lysis, cDNA was synthesized and amplified. An Illumina sequencing library was then prepared with the amplified cDNA. The resulting library was sequenced using a custom program on the Illumina NovaSeq 6000.

### Sequencing

Twenty-eight bp of cell barcode and UMI sequences and 91 bp RNA reads were generated with the Illumina NovaSeq 6000 at the Center for Medical Genomics of Indiana University School of Medicine, Indianapolis, IN.

### Analysis of scRNAseq data

CellRanger 3.1.0 (http://support.10xgenomics.com/) was utilized to process the raw sequence data generated using bcl2fastq (https://support.illumina.com/) to demultiplex raw base sequence calls into sample-specific FASTQ files. The FASTQ files were aligned to the zebrafish reference genome Danio_rerio.GRCz11 with RNAseq aligner STAR. The aligned reads were traced back to individual cells and gene expression levels were quantified based on the number of UMIs (unique molecular indices) detected in each cell. The filtered feature-cell barcode matrices generated by CellRanger were used for further analysis.

### Seurat analysis

The R package Seurat development version 3.1.0 [15–17] was used for cell type/state discovery with graph-based clustering, cell cluster marker gene identification, and various visualizations. Quality control (QC) metrics of library size, number of features/genes, and mitochondrial reads (based on median-absolute-deviation (MAD), 3 MAD used here) were calculated with scater [18]. An integrated comparative analysis of 2 single cell datasets (UPEC vs saline) was performed using Seurat development version 3.1.0 [15–17, 19]. The unwanted cell-cell variation in gene expression driven by experimental batch, cell alignment rate, the number of detected molecules, and mitochondrial gene expression was removed using a linear model. Cells were clustered using the principle component analysis (PCA) and visualized using the T-Distributed Stochastic Neighbor Embedding (t-SNE) plot. The gene markers for each cluster were identified through differential expression analysis by comparing cells in the cluster to all other cells. Clusters were identified with the Seurat functions “FindNeighbors” and “FindClusters” using a resolution of 0.9 and 36 principal components (PCs).

### Quality control

Low quality cells were excluded with the following criteria: cells with unique feature/gene counts over 4,000 or less than 300, or >20% mitochondrial genes.

### Data analysis

The FindConservedMarkers function identified conserved canonical cell type marker genes This function performs differential gene expression for each group and combines the *P* values using meta-analysis methods from the MetaDE R package. We started with the 9 most differentially expressed genes, but if these were indeterminate we expanded the search range. We also compared cluster expression to past zebrafish single cell transcriptomics [20]. The cell's predicted location of origin was identified by review of the Zfin database and the cluster assigned [21]. Differences of gene expression in a same cell cluster in the UPEC vs saline groups were identified using the “FindMarkers” function for cells between different clusters. A *P* value <0.05 was used as a cutoff with the significance presented as an adjusted *P* value.

### RNA velocity analysis

The mapping position sorted .bam files generated by 10x cellRanger pipeline were converted to cellsorted.bam files using samtools software version 1.9 in python version 3.6.8. Then .loom files were generated with velocyto-run command in python command line interphase (CLI) to generate spliced/unspliced expression matrices file. Finally RNA velocity vector projection was done with velocyto.R using an individual .loom file and integrated seurat object file (.RDS) and pagoda2 library function.

### Ingenuity canonical pathway analysis

Ingenuity pathway analysis (IPA) (Qiagen) was performed to determine the changes in transcriptome of cell subsets upon UPEC infection. Pathways were filtered by –log(*P* values) >1.3 and changes in z-score upon UPEC infection (negative z-score predicts downregulation, positive z-score predicts upregulation). The top 10 pathways with the highest z-score were presented.

## RESULTS

### scRNAseq

In the saline group, 2810 cells passed the criteria for inclusion. There was a mean of 197,958 reads per cell and a median of 1,004 genes per cell. In the UPEC group 21,830 cells met the criteria for inclusion, there were a mean of 22,145 reads per cell and a median of 711 genes per cell. A summary of the saline and UPEC group scRNAseq results can be found in Supplemental material 1. The source data for the scRNAseq can be found at the National Center for Biotechnology Information (NCBI) Gene Expression Omnibus (GEO) at https://www.ncbi.nlm.nih.gov/geo/query/acc.cgi?acc=GSE160038.

**Figure 1:**
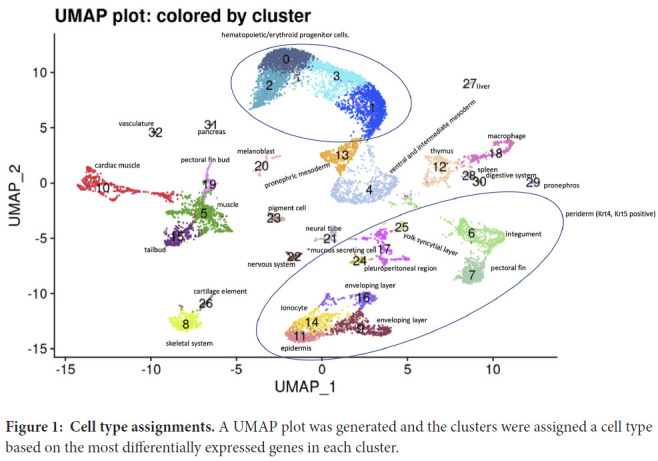
**Cell type assignments.** A UMAP plot was generated and the clusters were assigned a cell type based on the most differentially expressed genes in each cluster.

On integrated saline and UPEC group clustering analysis, 33 clusters were identified. Cluster assignments are presented as a uniform manifold approximation and projection (UMAP) plot in Figure 1, with the genes used to assign clusters presented in Supplemental material 2.

### Confirmation of infection model

**Figure 2:**
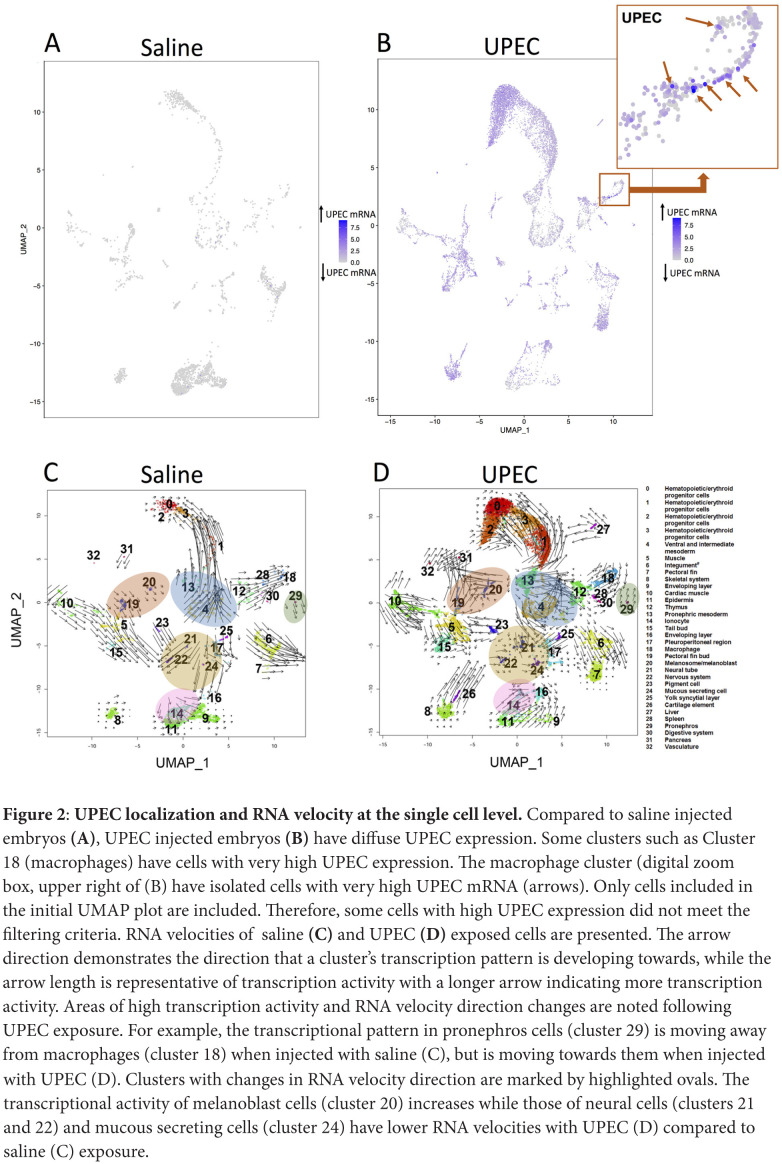
**UPEC localization and RNA velocity at the single cell level.** Compared to saline injected embryos **(A)**, UPEC injected embryos **(B)** have diffuse UPEC expression. Some clusters such as Cluster 18 (macrophages) have cells with very high UPEC expression. The macrophage cluster (digital zoom box, upper right of (B) have isolated cells with very high UPEC mRNA (arrows). Only cells included in the initial UMAP plot are included. Therefore, some cells with high UPEC expression did not meet the filtering criteria. RNA velocities of saline **(C)** and UPEC **(D)** exposed cells are presented. The arrow direction demonstrates the direction that a cluster's transcription pattern is developing towards, while the arrow length is representative of transcription activity with a longer arrow indicating more transcription activity. Areas of high transcription activity and RNA velocity direction changes are noted following UPEC exposure. For example, the transcriptional pattern in pronephros cells (cluster 29) is moving away from macrophages (cluster 18) when injected with saline (C), but is moving towards them when injected with UPEC (D). Clusters with changes in RNA velocity direction are marked by highlighted ovals. The transcriptional activity of melanoblast cells (cluster 20) increases while those of neural cells (clusters 21 and 22) and mucous secreting cells (cluster 24) have lower RNA velocities with UPEC (D) compared to saline (C) exposure.

We screened the single cell results for UPEC expression as confirmation of our infection model and to see if the UPEC segregate to distinct clusters. The data of the analysis shows non-homogenous UPEC gene distribution suggesting that zebrafish were systemically infected with UPEC (Figure 2A-B). Also zebrafish were injected with RFP-UPEC. After 24 hours, the zebrafish were dissociated and then homogenate was placed on a glass slide and evaluated for RFP expression. The confirmation of infection is demonstrated in Supplemental material 3.

### RNA velocity projection

Single cell sequencing captures the static snapshot of RNA abundance at a point in time, while RNA velocity is a high-dimensional vector that predicts the future state of individual cells [22]. Using RNA velocity, gene expression state can be directly estimated by distinguishing between unspliced and spliced mRNAs. This analysis showed several key changes in the transcriptional state when applied to the cells in this study (Figure 2C-D). Some clusters had a reversal of RNA velocity direction indicating that the cluster's mRNA expression pattern changed more consistently with a different neighboring cluster after exposure to UPEC. Also, the effect of UPEC on transcriptional activity is heterogenous between clusters, with some clusters having increased activity, some having decreased activity, and others having similar activity when compared to saline-exposed cells.

### Differentially expressed genes

We recorded genes that were upregulated or downregulated by at least 2.5-fold following UPEC exposure with a *P* value of < 0.05. The top 20 significantly upregulated (ranked by adjusted P value) were reviewed and key innate immune genes are identified in Table 1. Innate immune functions of the identified genes involved mediation of cell proliferation, survival, protection from oxidative stress, and interaction with LPS. The complete list of differentially expressed genes is presented in Supplemental material 4.

**Table 1.** Key innate immune genes that are significantly differentially regulated (10 most upregulated or downregulated genes) in UPEC vs saline injected zebrafish embryos

Gene	Accession #^	Cluster/cell type	Average log FC/adjusted *P* value	*Innate immune role*	Ref
*Hsp-90ab1*	990415-95	0/hematopoetic 3/hematopoetic	1.91/5.89E-72 1.97/1.12E-50	Chaperones that maintain proper protein folding during cellular stress such as infection	[30]
*Hsp70.1* *Hsp70.2* *Hsp70.3*	990415-91 110405-1 110713-1	1/hematopoietic 13/pronephric mesoderm 13/pronephric mesoderm	−1.05/2.02E-09 −1.45/3.30E-03 −1.49/3.51E-3	Protects against cell death and bacterial toxin mediated inflammation	[31, 32]
*Wif1*	990712-17	5/muscle	−1.62/1.72E-04	Inhibiting WIF1 (Wnt inhibitory factor 1) promotes cell proliferation in response to bacterial infections	[33]
*Cxcl8b*	101026-3	6/integument	−2.01/7.25E-44	Chemotaxis and inflammatory signaling	[34]
*Foxo1a*	061013-59	14/ionocyte 11/epidermis	−1.37/2.93E-13 −1.14/1.62E-43	Mediates lipopolysaccharide (LPS)-induced cytokine expression	[27]
*Arg2*	030131-1334	14/ionocyte 11/epidermis	−1.27/2.71E-14 −1.45/3.04E-50	Binds bacterial lipopolysac-charide	[35]
*Trib3*	040426-2609	14/ionocyte	−1.11/3.94E-15	Regulator of TLR2 mediated signaling in response to lipopolysaccharide	[29]
*Ccl20a.3*	081022-193	17/pleuroperitoneal region	2.16/1.45E-02	Salt sensitive antimicrobial activity against bacteria at low concentrations	[36]
*Rpl22l1*	081022-193	18/macrophages 4/mesoderm 10/cardiac muscle	1.2/4.69E-7 1.33/2.58E-57 1.6/1.85E-20	Potentially involved in production of mitochondrial proteins during bacterial phagocytosis by macro-phages	[37]
*Prdx2*	030326-2	22/nervous system	2.21/2.23E-26	Protects against oxidative stress	[38]
*Odcl*	10816-1	6/integument	−1.48/4.68E-65	Inhibits inflammatory response and ROS-induced apoptosis	[39]
*Mmp13b*	030131-6152	7/pectoral fin	−1.96/3.5E-8	Involved with epithelial cell migration in response to infection	[40]
*Plk3*	030131-6152	8/skeletal system	−1.06/6.89E-19	Involved in LPS induced apoptosis, deleting this gene may be protective during sepsis	[41]
*Ppt1*	040426-2653	12/thymus	−1.32-1.61E-5	palmitoyl-protein thioester-ase 1 (Ppt1) is a lysosomal enzyme that affects a cells interaction with bacteria	[42]

^ from https://zfin.org

### Canonical pathway analysis

**Figure 3:**
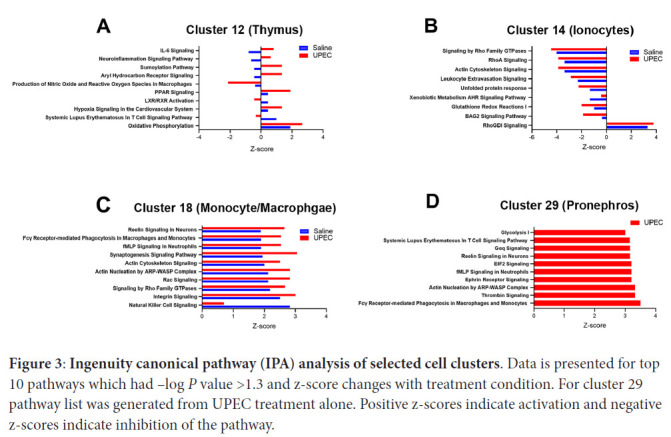
**Ingenuity canonical pathway (IPA) analysis of selected cell clusters.** Data is presented for top 10 pathways which had –log *P* value >1.3 and z-score changes with treatment condition. For cluster 29 pathway list was generated from UPEC treatment alone. Positive z-scores indicate activation and negative z-scores indicate inhibition of the pathway.

To determine the changes in the transcriptional activity of the genes within a cell cluster, ingenuity canonical pathway analysis was performed on selected cell clusters presumed to be important for innate immune response. Results of 4 cell clusters (12, 14, 18, 29) are presented in Figure 3. Primary themes in the leading pathway included response to LPS, stress protection, and immune cell migration and inflammatory signaling. The top 10 pathways were statistically significant -log (*P* value) >1.3 and had changes in z-score between treatment conditions (saline vs UPEC). Positive z-scores reveal up-regulation while negative z-scores reveal down-regulation of the pathway. The gene expression profiles that provided the basis for key pathway rankings are presented as Supplemental material 5.

## DISCUSSION

Zebrafish have been used previously as an infection model [23]. Here we used zebrafish embryos injected with uropathogenic *E.coli* (UPEC) as an experimental model to study bacterial infection at the single cell level. With past zebrafish models, pericardial injections caused local infections and duct of Cuvier or venous injections caused systemic infections [24]. We identified bacteria across cell types (Figure 2A-B), therefore, our injection method either involves dissemination of infection from the pericardial space or included injection into the venous circulation and/or remnant duct of Cuvier. Because zebrafish do not generally develop an adaptive immune response in the first month following fertilization, we could focus on the innate immune system by use of ~4-day post fertilization larvae [9].

scRNAseq allows the gene expression profile to be identified at the single cell level. scRNA-seq has advantages which includes allowing evaluation of rare cell types and heterogeneity within cell types. For example, in macrophages we were able to show heterogenous cells with high UPEC mRNA (Figure 2). We have used flow or magnetic antibody sorting to perform bulk RNAseq studies in mice, but antibodies are less readily available for zebrafish [25].

Our results show that several innate/immune genes were significantly upregulated and downregulated in the infected cohort. A subset of these genes with known innate immune function are discussed in Table 1. Lipopolysaccharide (LPS) is a primary endotoxin of UPEC and several genes significantly downregulated in UPEC injected embryos were involved in innate immune and/or inflammatory signaling secondary to LPS [26]. For example, Forkhead Box O1a *(Foxo1a)* and Tribbles Pseudokinase 3 (*Trib3)* mediate the cytokine expression and toll like receptor 2 signaling, respectively, in response to LPS [27–29]. Additionally, Arginase 2 (*Arg2)* binds LPS and Polo like kinase (*Plk3)* is involved in LPS induced apoptosis [29].

Therefore, at the timepoint of the scRNAseq analysis in this study, the cellular response to LPS appeared to be downregulated. Bacterial suppression of the ideal cellular response or the cells are trying to limit excessive inflammation are both possibilities for these findings that warrant evaluation in future studies.

Some of the significantly downregulated genes in the scRNA-seq results would likely increase survival and/or promote recovery. For example, Polo-like kinase 3 (*Plk3*) is a serine/threonine kinase that contributes to apoptosis following excessive inflammation [41]. *Plk3* expression significantly decreases in muscle cells in the UPEC group, compared to the saline group. In mice, decreased *Plk3* expression is associated with reduced LPS-induced apoptosis reducing the likelihood of shock and mortality from LPS [41]. Decreased Wnt inhibitory factor 1 (*Wnt1*) which was also seen in the muscle cell cluster would be expected to increase WNT signaling. Wnt signaling has been demonstrated to be involved in a wide range of cellular processes related to bacterial infection including cell cycle control, cell migration, phagocytosis-related cytoskeleton reorganization and cell migration, apoptosis and autophagy [43].

We also identified cell types of interest and canonical pathway activation in the response to bacteremia. Cluster 14/ionocytes had multiple innate immune genes in its most significantly differentially expressed genes. Ionocyte-mediated innate immune pathways were significantly down-regulated upon UPEC infection except RhoGDI (guanine nucleotide-dissociation inhibitors). Ionocytes are epithelial cells that regulate acid and ion balance in the skin and gill of zebrafish [44]. Ionocytes have similarities to kidney intercalated cells which have innate immune defense roles [45]. Other cell types that primarily demonstrated increases in the z-score of leading canonical pathways in response to infection included thymus cells. Specifically, interleukin 6 (IL-6) signaling, production of nitric oxide and reactive oxygen species (ROS) were both activated while retinoid X receptor (RXR) activation was inhibited. IL-6 signaling is well known to have a central role in integrated bacterial defense and is being evaluated as a therapeutic target [46]. Perhaps the zebrafish is a good model system to study the effect of manipulating IL-6 without the covariate of an adaptive immune response. Reactive oxygen species production is being explored as a treatment for bacterial infection. RXR signaling is a primary controller of the inflammatory response and chemokine expression and we have shown this pathway to be important to the kidney's response to UPEC [25, 47]. In macrophages, Fcγ receptor-mediated phagocytosis was the second leading canonical pathway that increased in response to infection. Fc receptors, which are essential to the appropriate response to infection and can often be inhibitory or inflammatory may be critical to controlling phagocytosis and cytokine expression, so that it is sufficient for the organism to respond to the infection, but collateral damage from an excessive inflammatory response is limited [48]. Cluster 18 had some cells with concentrated UPEC mRNA indicating phagocytosis (Figure 2B), however, the embryos were not old enough to produce antibodies indicating that the genes that triggered the Fcγ receptor mediated phagocytosis pathway ranking might represent a form of antibody independent phagocytosis [49]. From our review of the literature we did not identify where key genes involved in Fcγ receptor-mediated phagocytosis in this analysis, such as *Rac1,* (Supplemental material 5) are also involved in antibody-independent phagocytosis [50].

This study did have limitations. The cell population with the highest numbers and most differentially expressed genes tended to be related to skin and skeletal systems. It is possible that our 24-hour timepoint represents a relatively late time point and earlier time points would be informative in future studies focused on the organs initially susceptible to UPEC infection including the kidney, intestine, spleen, and heart. Our cluster classifications are estimates, as more information becomes known about zebrafish single cell transcriptomics, some of the cluster assignments might need to be adjusted with future analysis. Future directions for our research include more in-depth analysis of upregulated and downregulated genes. Our current study was limited by relative paucity of cells in the saline control group. For example, in our RNA velocity analysis (Figure 2), the control (saline) group has a very low number of cells which passed our quality control when compared to the infected group, which resulted in a relatively low number of unspliced transcripts. Unfortunately, several of these cells were lost in the pipetting process. It would be beneficial to repeat this study with more saline control cells. Also, the zebrafish also lacks a bladder and lower urinary tract making it difficult to model cystitis and our injection model might have differences from an infection that ascends the urinary tract. Last, since we evaluated viable cells, some cells that are highly susceptible to UPEC and were not viable would not have been included.

In conclusion, we used a zebrafish infection model for single cell sequencing and demonstrated changes in transcription and RNA velocity with UPEC infection. Altering inflammatory signaling secondary to LPS and acid-base regulation might be key areas in which to use a zebrafish model to study interventions in future studies. Zebrafish are also being developed for model systems to study antibiotic efficacy and toxicities [51, 52]. In the future, zebrafish UPEC infection and antibiotic efficacy/toxicity models could be combined as an initial screen to guide treatments that warrant testing in higher vertebrates.

## SUPPLEMENTARY DATA

Supplementary data may be provided by the authors to benefit the reader. Supplementary data are not copyedited and are the sole responsibility of the authors. Questions or comments related to supplementary materials should be addressed to the corresponding author.

Supplemental material 1. Summary of scRNAseq results

Supplemental material 2. Cluster assignment by differentially expressed genes

Supplemental material 3: Confirmation of zebrafish infection model. Larvae homogenate was visualized from a group injected with UPEC (experimental) versus one that was not (control). The RFP expressing bacteria were clearly visible in the experimental group. The red fluorescent images were taken at the same exposure.

Supplemental material 4. Complete list of differentially expressed genes in each cluster

Supplemental material 5. Key genes that determine the canonical pathway assignments

The above listed supplementary materials are available at https://doi.org/10.6084/m9.figshare.16416504.v1.
