# Supplementary material for: A Pilot Single Cell Analysis of the Zebrafish Embryo Cellular Responses to Uropathogenic Escherichia coli Infection
Source: Pathog Immun. 2022 Feb 4;7(1):1–18. doi: 10.20411/pai.v7i1.479 (PMC8843076; doi:10.20411/pai.v7i1.479)
Supplement: Supplemental material 1. Summary of scRNAseq results [file pai-7-1-s01.pdf]

**Supplemental material S1**, summary of scRNA-seq results		
	Saline	UPEC
Cells	2,810	21,830
Genes per cell (median)	1,004	711
Fraction reads in cell	91.9%	87.9
Median UMI counts per cell	4,315	3,374
Total genes detected	23,149	25,610
Reads mapped to genome	91.5	95
Reads mapped confidently to genome	84.4	84.2
Reads mapped confidently to intergenic regions	6.8	7.5
Reads mapped confidently to intronic regions	7.0	6.4
Reads mapped confidently to exonic regions	70.5	70.3
Reads mapped confidently to transcriptome	67.2	67.2
Reads mapped antisense to gene	1.1	1.1
