# Supplementary material for: A Pilot Single Cell Analysis of the Zebrafish Embryo Cellular Responses to Uropathogenic Escherichia coli Infection
Source: Pathog Immun. 2022 Feb 4;7(1):1–18. doi: 10.20411/pai.v7i1.479 (PMC8843076; doi:10.20411/pai.v7i1.479)
Supplement: Supplemental material 2. Cluster assignment by differentially expressed genes [file pai-7-1-s02.pdf]

**Supplemental material S2. Cluster assignment by differentially expressed genes**					
Cluster	Assignment				
0	Hematopoietic/erythroid progenitor cells	*Kreg1*	*hbbe2*	*Hbae1.1*	*Hbae3*
1	Hematopoietic/erythroid progenitor cells	*Alas2*	*Cahz*	*Slc4a1a*	*Epb41b*
2	Hematopoietic/erythroid progenitor cells	*Hbae1.3.1*	*Alas2*	*Hbae3*	*Hbbe1.2*
3	Hematopoietic/erythroid progenitor cells	*Alas2*	*Cahz*	*Hbbe1.2*	*Hemgn*
4	Ventral and intermediate mesoderm	*Hmbsa*	*Mki67*	*Znfl2a*	
5	Muscle	Col6a1	Col6a2		
6	Integument#	*Col1a2*	*Aqp3a*	*Rbp4*	
7	Pectoral fin	*Fgfbp2b*	*Matn1*	*Col9a2*	*F13a1b*
8	Skeletal system	*Col2a1a*	*Ucmab*		
9	Enveloping layer	*Gbgt1l4*	*Anxa1c*	*Capn9*	*Ppl*
10	Cardiac muscle	*Tcap*	*Desma*	*Tnnc2*	
11	Epidermis	*Lgals1l1*	*Apoeb*	*Krt91*	*krtt1c19e*
12	Thymus	*Ccr9a*	*Cc9ra*	*Coro1a*	*Ccl38.6*
13	Pronephric mesoderm	*Ndrg1a*	*Cd63*		
14	Ionocyte^	*Atp6v1ba*	*Apt6v1g1*	*Rnaseka*	*Atp6ap1b*
15	Tail bud	*Fnla*	*Ifitm1*		
16	Enveloping layer	*Cldne*	*Tmem176l.3b*		
17	Pleuroperitoneal region	*Cldnh*	*Cldn15la*	*Gstp2*	
18	Macrophage	*Samsn1a*	*Ctss.2*		
19	Pectoral fin bud	*Ptx3a*	*Angptl6*	*And1*	*And2*
20	Melanosome/melanoblast	*Dct*	*Tyrp1b*	*Slc45a2*	*Tyrp1a*
21	Neural tube	*Elavl3*	*Gpm6aa*	*Stmn1b*	
22	Nervous system	*Mbpa*	*Mag*	*Tmem125b*	
23	Pigment cell	*Ednrba*	*ltk*	*Mlphb*	
24	Mucous secreting cell	*Clcn2c*	*Dmrt2a*		
25	Yolk syncytial layer	*Pla2g12b*	*Acsl5*	*Fabp1b.1*	*Apoe*
26	Cartilage element	*Matn*	*Cnmd*	*Snorc*	*Ucmab*
27	Liver	*Fabp10a*	*Apoc.1*	*Apoa1b*	*Ucp1*
28	Spleen	*Il4*	*Il18*		
29	Pronephros	*Pax2a*	*Foxj1a*	*Slc26a1*	*Hnfbb*
30	Digestive system	*Itln*	*Ccr9a*		
31	Pancreas	*Cela1.5a*	*Cpa4*	*Cpa5*	*Ctrb1*
32	Vasculature	*Sele*	*Myct1a*	*Lyve1a*	*Stab1*
