# Supplementary figures and images for: A Pilot Single Cell Analysis of the Zebrafish Embryo Cellular Responses to Uropathogenic Escherichia coli Infection
Source: Pathog Immun. 2022 Feb 4;7(1):1–18. doi: 10.20411/pai.v7i1.479 (PMC8843076; doi:10.20411/pai.v7i1.479)

**A**

Light microscopy

Control

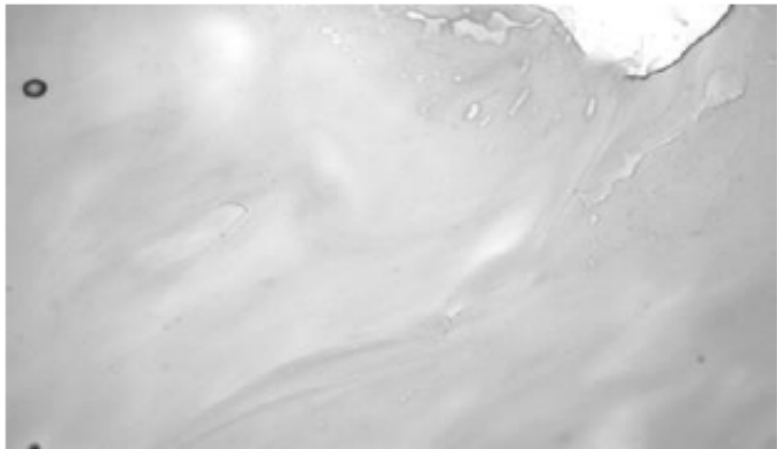**B**

Red fluorescence

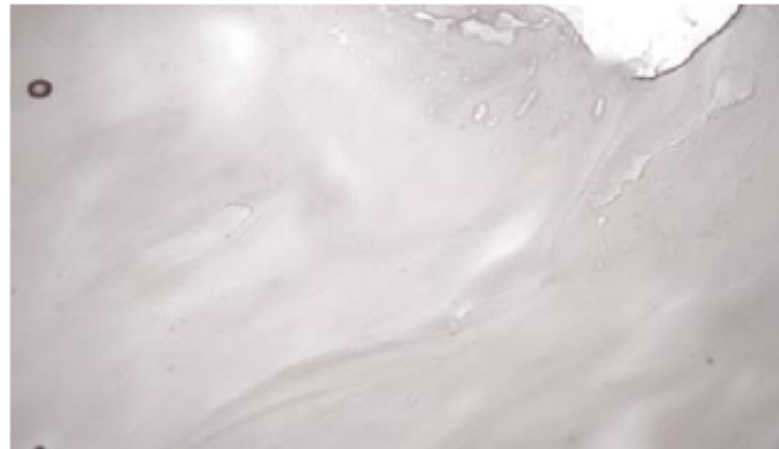

Experimental

**C**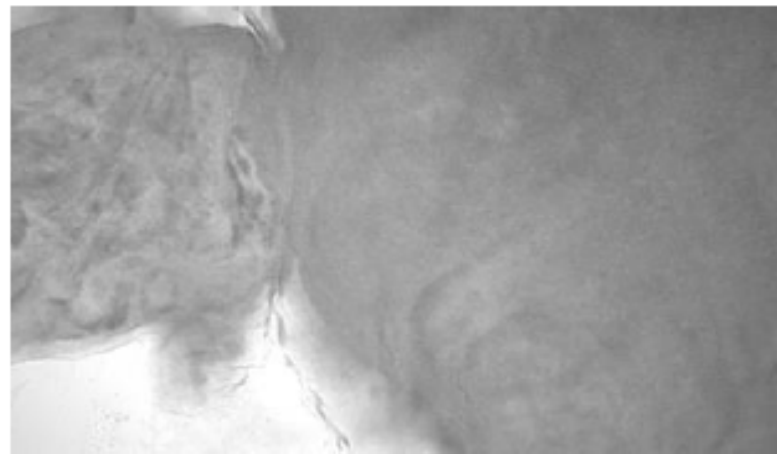**D**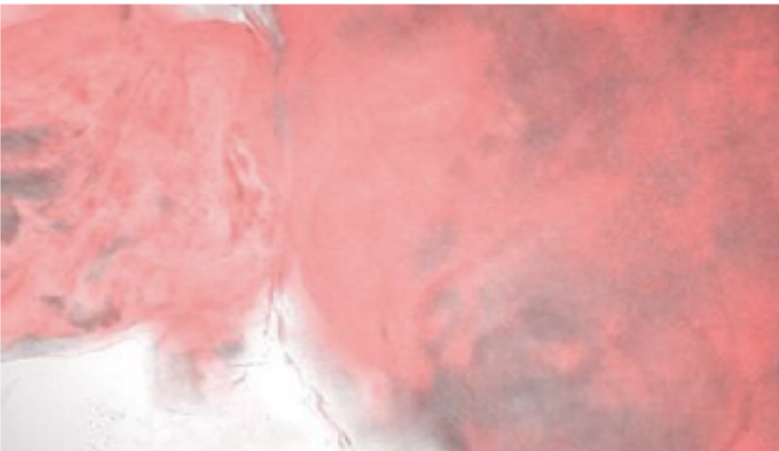

Supplement: Supplemental material 3: Confirmation of zebrafish infection model. Larvae homogenate was visualized from a group injected with UPEC (experimental) versus one that was not (control). The RFP expressing bacteria were clearly visible in the experimental group. The red fluorescent images were taken at  [file pai-7-1-s03.pdf]
